# [^18^F]FDG and [^18^F]FLT uptake in human breast cancer cells in relation to the effects of chemotherapy: an *in vitro* study

**DOI:** 10.1038/sj.bjc.6604523

**Published:** 2008-07-29

**Authors:** W G E Direcks, S C Berndsen, N Proost, G J Peters, J Balzarini, M D Spreeuwenberg, A A Lammertsma, C F M Molthoff

**Affiliations:** 1 Department of Nuclear Medicine & PET Research, VU University Medical Centre, P.O. Box 7057, 1007 MB Amsterdam, The Netherlands; 2 Department of Medical Oncology, VU University Medical Centre, P.O. Box 7057, 1007 MB Amsterdam, The Netherlands; 3 Rega Institute for Medical Research, Katholieke Universiteit Leuven, BE-3000 Leuven, Belgium; 4 Department of Clinical Epidemiology and Biostatistics, VU University Medical Centre, P.O. Box 7057, 1007 MB Amsterdam, The Netherlands

**Keywords:** [^18^F]FLT, [^18^F]FDG, breast cancer cells, thymidine kinase, PET, chemotherapy

## Abstract

Increased 2′-deoxy-2′-[^18^F]fluoro-D-glucose (FDG) uptake is the most commonly used marker for positron emission tomography in oncology. However, a proliferation tracer such as 3′-deoxy-3′-[^18^F]fluorothymidine (FLT) might be more specific for cancer. 3′-deoxy-3′-[^18^F]fluorothymidine uptake is dependent on thymidine kinase 1 (TK) activity, but the effects of chemotherapeutic agents are unknown. The aim of this study was to characterise FDG and FLT uptake mechanisms *in vitro* before and after exposure to chemotherapeutic agents. The effects of 5-fluorouracil (5-FU), doxorubicin and paclitaxel on FDG and FLT uptake were measured in MDA MB231 human breast cancer cells in relation to cell cycle distribution, expression and enzyme activity of TK-1. At IC_50_ concentrations, 5-FU resulted in accumulation in the G1 phase, but doxorubicin and paclitaxel induced a G2/M accumulation. Compared with untreated cells, 5-FU and doxorubicin increased TK-1 levels by >300. At 72 h, 5-FU decreased FDG uptake by 50% and FLT uptake by 54%, whereas doxorubicin increased FDG and FLT uptake by 71 and 173%, respectively. Paclitaxel increased FDG uptake with >100% after 48 h, whereas FLT uptake hardly changed. In conclusion, various chemotherapeutic agents, commonly used in the treatment of breast cancer, have different effects on the time course of uptake of both FDG and FLT *in vitro*. This might have implications for interpretation of clinical findings.

Breast cancer is the most frequently occurring cancer in women in the Western world. About 30% will develop metastases and may die of the disease (Weigelt *et al*, 2005). These patients may receive neo-adjuvant chemotherapy, but only a minority will respond. Selecting nonresponders would prevent them from unnecessary toxicity and offer a means to modify therapeutic strategy or to revert to palliative treatment. Conventional imaging technologies such as US, CT and MRI are unable to identify response at an early stage, as they primarily detect anatomical changes. Positron emission tomography (PET) is a noninvasive functional imaging technique that allows for the measurement of molecular processes and that could be very valuable for monitoring response early during treatment. 2′-deoxy-2′-[^18^F]fluoro-D-glucose (FDG) is already commonly used for this purpose, but the role of 3′-deoxy-3′-[^18^F]fluorothymidine (FLT) still needs to be evaluated.

2′-deoxy-2′-[^18^F]fluoro-D-glucose is transported into the cell by the same (glucose) transporters as glucose and is phosphorylated by hexokinases (HKs). In contrast to glucose-6-phosphate, FDG-6-phosphate is not a substrate for the glycolytic pathway, resulting in cellular trapping of ^18^F-labelled FDG-6-phosphate. Warburg *et al* (1927) already reported that often glucose metabolism is enhanced in tumours. To date, FDG PET is widely used for tumour detection and staging, and for response monitoring (Shields, 2006). 2′-deoxy-2′-[^18^F]fluoro-D-glucose uptake, however, is not specific for tumours. High physiological glucose consumption, and consequently high FDG uptake, can also be observed in brain, muscle and inflammatory tissues (Maschauer *et al*, 2004).

3′-deoxy-3′-[^18^F]fluorothymidine, a thymidine analogue, was suggested as a tracer for cell proliferation (Shields *et al*, 1998). 3′-deoxy-3′-[^18^F]fluorothymidine is phosphorylated by the cytosolic enzyme thymidine kinase 1 (TK-1) into FLT-monophosphate, which is trapped in the cell (Direcks *et al*, 2006). High levels of TK-1 are found in proliferating and malignant cells and its activity increases with DNA synthesis and in the S-phase (Munch-Petersen *et al*, 1991), but is low in nondividing cells (Sherley and Kelly, 1988). Another isoform of TK is TK 2 (TK-2), predominantly localised in mitochondria, present in nonproliferating cells (Eriksson *et al*, 2002). In contrast to thymidine, FLT is poorly incorporated into DNA (Sundseth *et al*, 1996). 3′-deoxy-3′-[^18^F]fluorothymidine uptake is correlated with S-phase (Rasey *et al*, 2002), Ki67 immunostaining (Kenny *et al*, 2005) and TK-1 levels (Barthel *et al*, 2005). As, anticancer treatment can inhibit proliferation it may change FLT uptake, implying a role for FLT as a marker for monitoring response to chemotherapy.

Chemotherapeutic agents with different modes of action, commonly used in the treatment of breast cancer, are 5-fluorouracil (5-FU), doxorubicin and paclitaxel (Honkoop *et al*, 1999). 5-fluorouracil is metabolised and one of the metabolites is an inhibitor of thymidylate synthase (TS), a key enzyme in the *de novo* pathway, which provides thymidine for DNA synthesis (Ackland *et al*, 2002). Doxorubicin induces DNA damage by binding to the nuclear protein topoisomerase II, an enzyme important for correcting DNA geometry during transcription and replication. Single- and double-DNA strand breaks are induced and DNA synthesis is inhibited (Potter and Rabinovitch, 2005). Paclitaxel belongs to the group of taxanes that stabilise microtubuli, including those in the mitotic spindle, thereby blocking cell division and inducing apoptosis (Kavallaris *et al*, 2001).

The purpose of this study was to investigate the effect of these chemotherapeutic agents on FDG and FLT uptake in human breast cancer cells *in vitro* and to relate the results with biological parameters, such as TK-1 expression, and activity and cell cycle distribution. Insight into these molecular mechanisms should assist in interpreting FDG and FLT results when monitoring response to chemotherapy in a clinical setting.

## Materials and methods

### Cell lines

The human breast cancer cell line MDA MB231 (ATCC no. HTB-26) and CEM leukaemia cells (CEM wild type and TK-deficient CEM/TK-) were cultured in RPMI 1640 supplemented with 10% foetal bovine serum and 1% penicillin/streptomycin (P/S), at 5% CO_2_ in a humidified atmosphere at 37°C. Cell counting was performed with a Casy cell counter (Schärfe System GmbH, Reutlingen, Germany).

### Cytotoxicity assay

Cytotoxicity to 5-FU (Sigma Chemical Co., MO, St Louis, USA), doxorubicin (doxorubicin hydrochloride, Pfizer, Cappelle a/d IJssel, The Netherlands) and paclitaxel (Sigma Chemical Co.) was determined by sulforhodamine (SRB) assay. Briefly, cells were plated in 96-well plates and after 24 h, drugs were added at various concentrations, incubated for 72 h and the SRB assay was performed as described previously (de Bruin *et al*, 2003). The IC_50_ is the concentration resulting in 50% reduction in growth compared with untreated control cells.

### Cell cycle analysis

For cell cycle analysis, cells were exposed to IC_50_ concentrations of the various drugs for 4, 24, 48 or 72 h, fixed in 70% ethanol (1 × 10^6^ cells per ml) and stored at 4°C until analysis, as described previously (Temmink *et al*, 2007) according to a slightly modified protocol.

### Production of PET tracers

2′-deoxy-2′-[^18^F]fluoro-D-glucose at a radiochemical purity of >97% was produced by BV Cyclotron VU (Amsterdam, The Netherlands).

3′-deoxy-3′-[^18^F]fluorothymidine was synthesised according to a modified procedure originally described by Machulla *et al* (2000). This procedure resulted in a GMP compliant, pyrogen free, sterile production of FLT with a radiochemical purity >97%, an average yield of 1.5±0.5 GBq and a mean specific activity of 93±33 GBq *μ*mol^−1^.

### Cell extract preparation

Cells were plated in 75 cm^2^ flasks and, after 24 h, exposed to IC_50_ drug concentrations for 4, 24, 48 or 72 h, after which the cells were harvested, spun down and snap-frozen into liquid nitrogen. Cell pellets were stored at −80°C until use.

### TK enzyme activity

Thymidine kinase-1 and -2 enzyme activities were measured using thymidine as a substrate, as described previously (van der Wilt *et al*, 2001). Enzyme activity was also analysed in CEM cells and in the corresponding TK-1-deficient subtype to assess whether FLT phosphorylation only occurs in the presence of sufficient cytosolic TK-1.

Cell pellets were suspended in 50 mM Tris/1 mM EDTA (pH 7.4) and sonificated on ice. Lysates were spun down and supernatant diluted 1 : 8 in 50 mM Tris/1 mM EDTA (pH 7.4).

3′-deoxy-3′-[^18^F]fluorothymidine was diluted with 50 mM Tris/1 mM EDTA (pH 7.4) and 1 : 1 mixed with 20 mM ATP, 10 mM MgCl_2_, resulting in an FLT concentration of about 60 MBq ml^−1^ (6.32–18.89 pmol per sample). Thymidine kinase-1 was inhibited by addition of dCTP or the specific TK-2 inhibitor KIN52 (final concentration 2 *μ*M) (Balzarini *et al*, 2003).

The assay was optimised for protein concentration and incubation times. Diluted cell lysates or purified TK-2 (kindly provided by A. Karlsson, Karolinska Institute, Stockholm, Sweden) were incubated with the FLT mixture for 15 min at 37°C and the reaction was stopped by heating the samples at 95°C for 5 min. Substrate (FLT) and product (FLT-phosphate) were separated by TLC and radioactivity was measured in a single-well gamma counter (Wallac1480 Wizard, Perkin Elmer Lifescience, MA, USA). Protein concentrations were measured in a Bio-Rad Bradford protein assay (Bio-Rad Laboratories, Hercules, CA, USA). Thymidine kinase activity was calculated as the amount of FLT converted into FLT-phosphate per hour per million cells (nmol h^−1^ 10^−6^ cells).

### Affinity of FLT, KIN52 and dCTP for TK

IC_50_ of KIN52 and dCTP against phosphorylation of 1 *μ*M [CH_3_-^3^H][dThd] as natural substrate for recombinant cytosolic TK-1 and mitochondrial TK-2 were determined as described below. A 50 *μ*l reaction mixture, containing 50 mM Tris-HCl, pH 8.0, 2.5 mM MgCl_2_, 10 mM dithiothreitol, 2.5 mM ATP, 1.0 mg ml^−1^ bovine serum albumin, 10 mM NaF, [CH_3_-^3^H]dThd (0.1 *μ*Ci in 5 *μ*l; 1 *μ*M final concentration) and 5 *μ*l of recombinant enzyme, was incubated at 37°C for 30 min in the presence or absence of different concentrations of dCTP or KIN52. During this time, the enzyme reaction proceeded linearly. Formation of tritiated dTMP was measured by spotting aliquots of the reaction mixture on Whatman DE-81 filter paper disks, as described earlier (Balzarini *et al*, 2003).

### TK-1 levels

For detection of TK-1 protein expression, cell pellets were dissolved in lysis buffer containing 10 mM Tris/5 mM EDTA (pH 7.5), 10% glycerol, 150 mM NaCl, 50 mM *β*-mercapto-ethanol, 1% Triton X-100, 4% protease inhibitor cocktail (PIC, Boehringer, Germany) and 1 mM NaVO_3_, sonificated on ice, spun down and the supernatant stored at −80°C until use. Lysates were analysed with 12% SDS–PAGE followed by blotting onto a polyvinylidene difluoride membrane. Thymidine kinase-1 was detected by overnight incubation with 1 : 150 dilution of an anti-TK-1 monoclonal antibody (QED Bioscience, San Diego, CA, USA) followed by 60 min incubation with a 1 : 2000 dilution of secondary antibody (horseradish peroxidase-conjugated rabbit anti-mouse antibody, DakoCytomation, Glostrup, Denmark) as described previously (Temmink *et al*, 2007). *β*-actin was used to control for loading.

### Uptake of FDG and FLT

Cells were plated in six identical 24-well plates at 75 000 cells per ml. After 24 h, IC_50_ drug concentrations (5-FU, doxorubicin and paclitaxel) were added. Control cells were plated in a concentration of 45 000 cells per ml, 4 days before tracer uptake. Cell culture medium was replaced 4 h before adding PET tracers. For FDG plates, medium was replaced with medium without glucose (+IC_50_ drugs concentration), as glucose competes with FDG uptake. For FLT plates, medium was replaced with medium without L-glutamine (+IC_50_ drugs concentration). The presence of L-glutamine in the culture medium may reduce FLT uptake, as glutamine is a source of thymidine monophosphate competing with FLT uptake through the *de novo* pathway.

One MBq per well FDG or FLT was added to the cells and incubated for 60 min at 37°C. After removal of excess tracer, cellular tracer uptake was determined with an ECAT Exact HR+ PET scanner (Siemens/CTI, Knoxville, TN, USA). Images were analysed using CAPP software (version 7.2, CTI/Siemens, Knoxville, TN, USA). Regions of interest (ROI) were drawn manually in a plane with visible tracer uptake. Next, ROIs were copied to all other planes and total activity per well was calculated and plotted against exact number of cells. Tracer uptake was also determined in an identical plate using the single-well gamma counter. Tracer uptake was corrected for the number of cells.

To discriminate between FLT and FLT-phosphate, cells in another identical plate were harvested and after stopping the enzyme reaction, cells were sonificated and spun down. 3′-deoxy-3′-[^18^F]fluorothymidine and FLT-phosphate, present in the supernatant were separated as described above (section TK enzyme activity).

### Statistics

The longitudinal relation of drug incubation (untreated cells, 5-FU, doxorubicin, paclitaxel) on cell cycle phase, TK activities and levels, and on FDG and FLT uptake was analysed using GEE analyses, taking into account that the same cell line was measured repeatedly and by using all available data, irrespective of the number of repeated measurements. The GEE analysis is capable of dealing with irregular time intervals and corrects for the dependency of observations by adding a ‘within subject correlation structure’ to the regression model (Twisk, 2006). An exchangeable correlation structure was used, which means that correlations between subsequent measurements are assumed to be the same, irrespective of the time between measurements. Three dummy variables indicating the various drugs (untreated cells as reference category) and four dummy variables indicating time and interaction between drug and time were used as independent variables. Differences between the various drugs were compared at each incubation time. Before analysis, a logistic transformation of the data was performed. Statistical analyses were performed using SPSS version 15.0. *P*-values <0.05 were considered significant.

## Results

### Cytotoxicity assay

MDA MB231 cells were examined for their sensitivity to 5-FU, doxorubicin and paclitaxel, resulting in IC_50_ concentrations of 5 *μ*M, 200 and 2.5 nM, respectively. These concentrations were applied in the long-term cell culture experiments described in the following paragraphs.

### Cell cycle analysis

**Figure 1:**
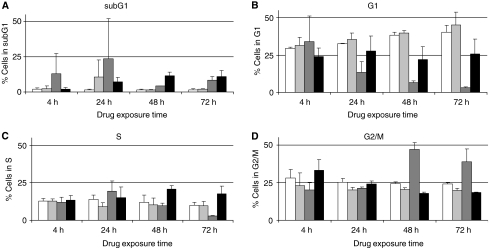
Distribution of MDA MB231 cells after exposure to IC_50_ concentrations of 5-fluorouracil (
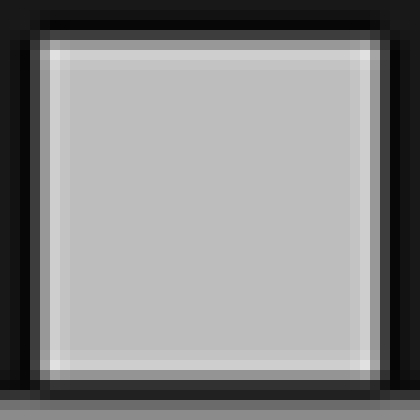
), doxorubicin (
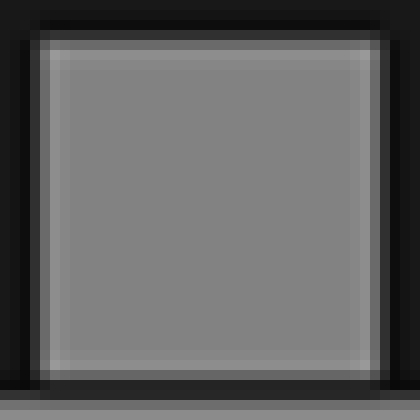
), paclitaxel (▪) and untreated cells (□), in (**A**) sub-G1, (**B**) G1, (**C**) S and (**D**) G2/M phases.

Cell cycle distribution was investigated at above-mentioned IC_50_ concentrations of 5-FU, doxorubicin and paclitaxel (Figure 1).

5-fluorouracil increased the percentage of cells in G1, whereas the number of cells in G2/M decreased compared with control cells, irrespective of incubation time (for statistics see below).

After 48 h incubation with doxorubicin, cells showed strong accumulation in the G2/M phase (two-fold). Meanwhile, 90 and 70% decreases in G1 and S phases, respectively, were observed. This cell cycle distribution pattern was already pronounced after 24 and 48 h incubation, respectively.

Paclitaxel exposure induced a 1.2-fold increase in G2/M phase at 4 h drug incubation. After 48 and 72 h, paclitaxel induced increases in sub-G1 (representing also apoptotic cells) and S phases, whereas the percentage of cells in the G2/M decreased.

The GEE analysis demonstrated a significant difference for 5-FU in the G2 phase after 24 h (*β*=20.2, *P*<0.001). Doxorubicin incubation induced changes in the G2 phase that were significant for all incubation times (4 h: *β*=19.9, *P*<0.05; 24 h: *β*=21.3, *P*<0.01; 48 h: *β*=47, *P*<0.001; 72 h: *β*=38.3, *P*<0.001) and after 24 h in the G1 phase (24 h: *β*=12.4, 48 h: *β*=6.4, 72 h: *β*=3.1, *P*<0.001). For the S phase, only 72 h incubation showed a significant effect (*β*=2.9, *P*<0.0001). Paclitaxel incubation was significant in G2 and G1 phases after 48 h (G2: 48 h *β*=17.7, 72 h *β*=18, *P*<0.0001; G1: 48 h *β*=23.7, 72 h *β*=24.4, *P*<0.05). Comparable with doxorubicin, after paclitaxel incubation, the S phase was only significantly different after 72 h (*β*=17.1, *P*<0.01).

### TK enzyme activity

**Figure 2:**
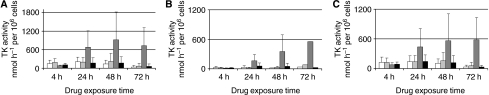
Thymidine kinase (TK) activities in MDA MB231 breast cancer cells (**A**) without, and with addition of the TK-2 inhibitors (**B**) dCTP and (**C**) KIN52. Cells were incubated with IC_50_ concentrations of 5-fluorouracil (
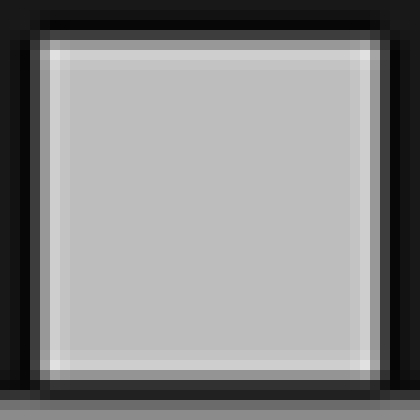
), doxorubicin (
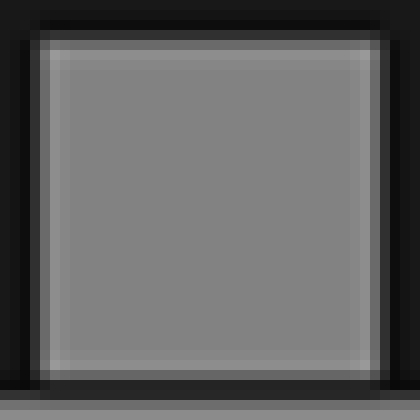
) and paclitaxel (▪), and compared with untreated cells (□).

For 5-FU and paclitaxel, increased TK activity compared with untreated cells was found at 48 and 72 h, but these effects were minor. MDA MB231 cells treated with doxorubicin showed more than three-fold increase in TK activity after 24 h and this effect was retained at longer incubation times (Figure 2A). The GEE analysis confirmed the findings after doxorubicin incubation as significant (24 h: *β*=550, *P*<0.05; 48 h: *β*=597, *P*<0.01; 72 h: *β*=602, *P*<0.0001).

**Figure 3:**
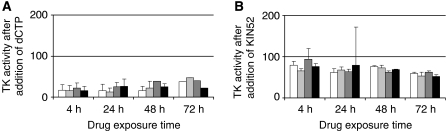
Relative thymidine kinase (TK)-1 activity in MDA MB231 breast cancer cells after addition of (**A**) dCTP and (**B**) KIN52. Cells were exposed to IC_50_ concentrations of 5-fluorouracil (
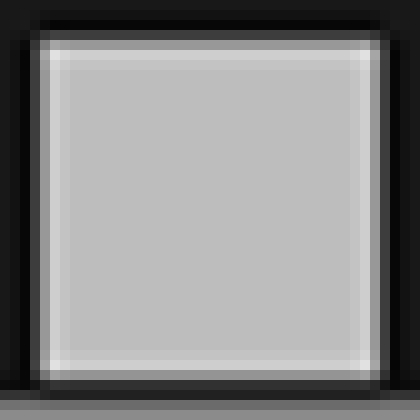
), doxorubicin (
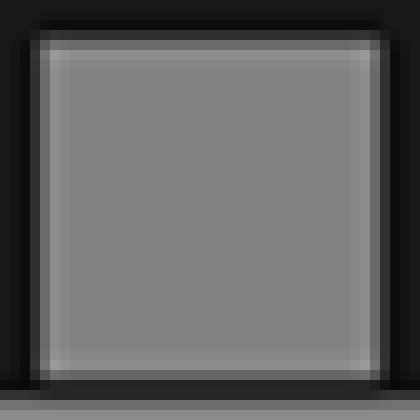
) and paclitaxel (▪), and compared with untreated cells (□). % cells are normalised to TK activity before addition of dCTP or KIN52 (set to 100%).

After adding the TK-2 inhibitors dCTP and KIN52 (10 and 2 *μ*M, respectively) to the cell lysates, remaining enzyme activity should primarily correspond to TK-1 (Figures 2B and C). Total TK activity was more strongly inhibited by dCTP than by KIN52 (Figures 3A and B): 55–90% of the cellular TK activities were inhibited by dCTP and 6–50% by KIN52. This may be related to the possibility that at the concentration used, dCTP also may inhibit TK-1 activity. Doxorubicin exposure resulted in a higher increase in TK-1 activity compared with untreated cells than exposure by 5-FU or paclitaxel.

**Figure 4:**
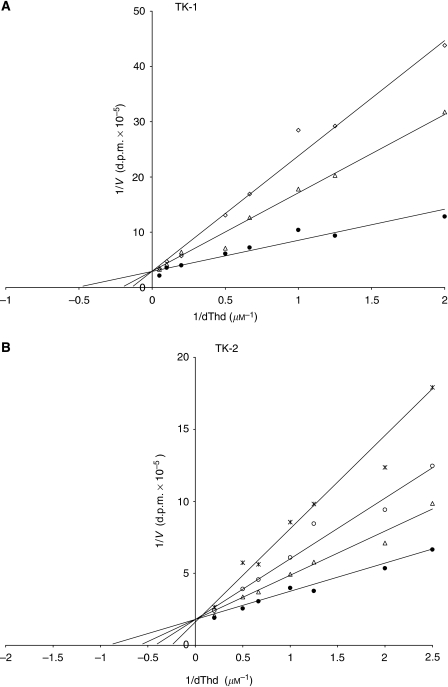
Lineweaver–Burk plots showing the effects of 3′-deoxy-3′-[^18^F]fluorothymidine (FLT) on thymidine (dThd) phosphorylation, catalysed by (**A**) thymidine kinase (TK)-1 at (▵) 25 *μ*M and (◊) 10 *μ*M, and (**B**) TK-2 at (▵) 2.5 *μ*M, (°) 5 *μ*M and (*) 10 *μ*M of FLT, compared with thymidine alone (•). dThd, thymidine; V, reaction rate.

The relative role of TK-2 in potential FLT phosphorylation was examined by adding FLT to purified TK-2. An 8% conversion of FLT into FLT-phosphate was detected implying that FLT was phosphorylated by TK-2. Lineweaver–Burk plots revealed a competitive inhibition of both TK-1- and TK-2-catalysed phosphorylation of dThd as the natural substrate (TK-1: Km_dThd_=2.3 *μ*M, Ki_FLT_=1.9 *μ*M, Ki/Km=8.5; TK-2: Km_dThd_=1.3 *μ*M, Ki_FLT_=4.2 *μ*M, Ki/Km=3.2) (Figures 4A and B), indicating that FLT had affinity for TK-2. When the effect of FLT on TK-1-mediated dThd phosphorylation was evaluated, the specific TK-2 inhibitors dCTP (10 mM) and KIN52 (2 *μ*M) inhibited FLT phosphorylation by TK-2 with 97 and 90%, respectively, implying that dCTP at this concentration is a more effective TK-2 inhibitor. KIN52 had an IC_50_ of 1.3±0.1 *μ*M against TK-2, as compared with >100 *μ*M against TK-1. When dCTP was evaluated at different concentrations for its inhibitory effect against TK-1 and TK-2, a dose-dependent inhibition of both enzymes was observed, TK-2 being inhibited more efficiently (IC_50_: ∼50 *μ*M) than TK-1 (IC_50_: ∼5 *μ*M).

### TK-1 levels

**Figure 5:**
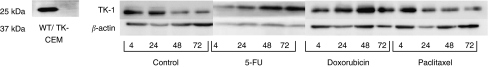
Typical Western blot for thymidine kinase (TK)-1 levels in CEM and MDA MB231 cells after treatment with cytotoxic agents compared with untreated cells, *β*-actin was used as loading control.

As enzyme activity is determined, at least in part, by the amount of TK-1 and TK-1 activity may be different for FLT compared with, for example, thymidine, protein levels in MDA MB231 cells were also analysed after treatment with IC_50_ drug concentrations. Thymidine kinase exists in different conformations and after Western blotting using MDA MB231 cells, both a dimer and a monomer were visible (data not shown). Using freshly prepared sample buffer and boiling of samples for at least 10 min resulted in complete denaturation of the dimer into the monomer (Figure 5). Furthermore, although TK-1 can also be phosphorylated (Chang *et al*, 1994), no antibodies were available to discriminate between these forms. Blots represent both phosphorylated and non-phosphorylated TK-1.

In general, 5-FU and doxorubicin treatment increased TK-1 levels nearly two-fold at 48 h (Table 1). Paclitaxel on the other hand induced a decrease of >40% in TK-1 levels at that time point. The GEE modelling demonstrated a significant difference in TK levels for paclitaxel incubation after 24 h (24 h: *β*=14, 48 h: *β*=7, 72 h: *β*=4, *P*<0.05) and for doxorubicin after 48 h (48 h: *β*=23, 72 h: *β*=33, *P*<0.005) compared with untreated cells. 5-fluorouracil incubated cells only demonstrated a significant difference in levels after 72 h (*β*=21, *P*<0.01).

As a control, TK levels were also assessed for CEM (Figure 5) and its TK-deficient subtype. CEM WT had a high TK-1 level, but no detectable TK was found in TK-deficient cells.

### Cellular FDG and FLT uptake

Uptake of FDG and FLT was determined in MDA MB231 cells after various incubation times with IC_50_ drug concentrations of 5-FU, doxorubicin and paclitaxel and results are summarised in Table 2. Cellular tracer uptake was determined with a PET scanner as well as a gamma counter and no differences were found between the two methods. The advantage of quantification using PET is that cells do not have to be transferred to another plate (or tube), possibly losing cells and thereby causing reduction of the signal and measurement errors.

5-fluorouracil incubation decreased FDG and FLT uptake by approximately 50% after 72 h incubation. Doxorubicin increased FDG uptake (>24%) at 24, 48 and 72 h, but increased FLT uptake irrespective of incubation time (>24%). Paclitaxel induced an increase in FDG uptake (>100%) at 48 and 72 h, and an increased FLT uptake (66%) at 72 h. The GEE showed a significant difference in FDG uptake after 5-FU incubation, irrespective of incubation time (4 h: *β*=0.79, 24 h: *β*=0.82, 48 h: *β*=0.80, 72 h: *β*=0.43, *P*<0.005) and after 48 h of paclitaxel incubation (48 h: *β*=0.94, 72 h: *β*=1.0 *P*<0.001). For 5-FU, FLT uptake after 72 h incubation was significant according to GEE (72 h: *β*=0.04, *P*<0.005), whereas changes were already significant after 48 h incubation for doxorubicin (48 h: *β*=0.17, 72 h: *β*=0.15, *P*<0.005) and paclitaxel (48 h: *β*=0.07, 72 h: *β*=0.10, *P*<0.0001).

One hour after adding FLT, cells were disrupted and cell lysates were loaded on a TLC plate to separate FLT and FLT phosphate. In this case, more than 80% of cellular radioactivity counts were due to FLT phosphate, implying that less than 20% was unphosphorylated FLT.

## Discussion

The purpose of this study was to assess FDG and FLT uptake *in vitro* in human breast cancer cells in relation to the effects of chemotherapy. Insight into molecular mechanisms involved in tracer uptake and retention will provide directions for interpretation how these tracers can be used to monitor chemotherapy in a clinical setting. Changes in tracer uptake were related to TK enzyme levels after exposure to three different chemotherapeutic agents.

In MDA MB231 human breast cancer cells, exposure to 5-FU decreased FDG uptake, possibly related to a decreased activity of either the glucose transporter Glut-1 or the phosphorylation enzyme HK. In MCF-7 breast cancer cells, increased Glut-1 mRNA levels were found in the first 24 h of 5-FU and doxorubicin treatment, whereas Glut-1 protein levels in doxorubicin-treated cells decreased (Engles *et al*, 2006). Also, [^3^H]FDG uptake decreased after both treatments, parallel to a decline in HK II mRNA levels (Engles *et al*, 2006). In this study, also decreased FDG uptake after 5-FU and within the first 24 h of doxorubicin treatment was found. 3′-deoxy-3′-[^18^F]fluorothymidine uptake in 5-FU exposed MDA MB231 cells was also decreased. Although cells showed a G1 arrest (where TK-1 activity should be low), increased TK protein levels were found, but enzyme activity remained similar or increased much less compared with TK-1 levels in untreated cells. Dittmann *et al* (2002) reported an S-phase arrest after 5-FU using OSC-1 oesophageal squamous carcinoma cells (where TK levels and activity should be high), whereas Mirjolet *et al* (2002) reported G1/S phase accumulation rather than a G0/G1 accumulation due to 5-FU cytotoxicity. Above reports are highly indicative of different effects of 5-FU, depending on the cell line. Decreased FLT uptake could be explained by differences in thymidine providing pathways in the cell. 5-fluorouracil inhibits the *de novo* TMP synthesis pathway. The salvage pathway, however, facilitates FLT uptake and this pathway will be more activated when the *de novo* pathway is blocked, which might result in increased FLT uptake, as TS inhibition results in increased thymidine uptake and utilisation. Indeed, in radio-induced fibrosarcoma (RIF) and oesophageal carcinoma cells increased [^3^H]thymidine was observed after 6 h of 5-FU incubation (Dittmann *et al*, 2002; Yau *et al*, 2006). However, prolonged treatment (starting from 24 h onwards) resulted in decreased thymidine uptake in RIF-1, HT29 colon cancer cells (Yau *et al*, 2006) and C6 glioma cells (van Waarde *et al*, 2006), and in RIF tumours (Barthel *et al*, 2003). In addition, decreased TK levels in intestinal mucosal cells (Kralovanszky *et al*, 1993) have been reported. Both findings are in agreement with the present results in MDA MB231 cells.

Doxorubicin treatment induced a massive increase in FLT uptake, next to accumulation of cells in the G2/M phase. Doxorubicin binds to topoisomerase II, which is high in the G2/M phase. Consequently, DNA replication is no longer possible and cells are arrested in the G2/M cell cycle phase (Potter and Rabinovitch, 2005). It has been reported that in the G2/M phase, TK activity is high (Sherley and Kelly, 1988; Munch-Petersen *et al*, 1991), which is supported by the present results.

In this study, breast cancer cells incubated with paclitaxel showed an increase in FDG uptake. A G2/M arrest in paclitaxel incubated MDA MB231 cells was observed within 24 h of incubation, which is in agreement with other published data (Wang *et al*, 2000). 3′-deoxy-3′-[^18^F]fluorothymidine uptake after 72 h of paclitaxel incubation was slightly increased, probably due to increased TK activity in these cells. However, TK levels were decreased starting from 24 h incubation. So far, no other studies have been published on the effects of paclitaxel (or other taxanes) on uptake of thymidine (or thymidine analogues).

Controversy exists on whether FLT is a substrate for TK-2. Munch-Petersen *et al* (1991) reported that FLT is not phosphorylated by TK-2. In contrast, in this study, good affinity of FLT for (purified) TK-2 was found. The TK-2 inhibitors used, nearly blocked all of FLT-phosphorylation, although there was a difference between dCTP and KIN52, possibly due to the fact that dCTP may also inhibit TK-1 activity to some degree. Most of the TK activity in tumour cells is expected to be TK-1 activity, as tumour cells are (highly) proliferating and TK-1 is an S-phase-regulated enzyme. In contrast, TK-2 is predominant in nonproliferating cells (Eriksson *et al*, 2002). At present, however, it is not clear whether FLT could reach the mitochondria to be phosphorylated by TK-2 in intact tumour cells. The present results suggest that FLT is trapped in all cells, irrespective of the TK isoform being expressed.

Response monitoring early during chemotherapy is extremely valuable to select nonresponders as early as possible to prevent unnecessary toxicity. At present, PET is the most promising technique for early response monitoring. However, many potential selective (eg FLT) and less selective (eg FDG) PET tracers are available. More insight into cellular mechanisms involved in tracer uptake could aid in understanding and subsequent selection of the optimal tracer for a specific chemotherapeutic agent. In theory, *in vitro* studies such as those presented here could provide guidelines on expected effects *in vivo.* Further studies in patients should validate this concept.

## Figures and Tables

**Table 1:** Mean (±s.d.) change (%) in TK-1 protein levels of MDA MB231 cells following incubation with IC_50_ concentrations of 5-FU, doxorubicin and paclitaxel compared with untreated cells (set to 100%)

	**4 h**	**24 h**	**48 h**	**72 h**
5-FU	163±1	122±38	184±53	443±58
Doxorubicin	64±6	130±78	177±71	473±348
Paclitaxel	103±6	62±4	54±16	52±32

5-FU=5-fluorouracil; TK=thymidine kinase.

**Table 2:** Mean (±s.d.) change (%) in FDG and FLT uptake in MDA MB231 cells after incubation with IC_50_ concentrations of 5-FU, doxorubicin and paclitaxel compared with untreated cells (set to 100%)

	**5-FU**		**Doxorubicin**		**Paclitaxel**	
	**FDG**	**FLT**	**FDG**	**FLT**	**FDG**	**FLT**
4 h	67±2	99±45	57±35	123±83	105±41	108±2
24 h	70±13	113±7	124±59	137±52	125±99	112±54
48 h	69±21	101±8	133±45	494±232	207±163	118±14
72 h	45±20	46±32	171±0	273±209	228±182	166±18

FDG=2′-deoxy-2′-[^18^F]fluoro-D-glucose; FLT=3′-deoxy-3′-[^18^F]fluorothymidine; 5-FU=5-fluorouracil; TK=thymidine kinase.
